# Glucosinolate-Degradation Products as Co-Adjuvant Therapy on Prostate Cancer in Vitro

**DOI:** 10.3390/ijms20204977

**Published:** 2019-10-09

**Authors:** María Jesús Núñez-Iglesias, Silvia Novío, Carlota García, Elena Pérez-Muñuzuri, Pilar Soengas, Elena Cartea, Pablo Velasco, Manuel Freire-Garabal

**Affiliations:** 1 SNL Laboratory, School of Medicine and Dentistry, University of Santiago de Compostela, c/San Francisco, s/n, 15782 Santiago de Compostela, A Coruña, Spain; carlota.garcia@rai.usc.es (C.G.); mariaelena.perez@usc.es (E.P.-M.); manuel.freire-garabal@usc.es (M.F.-G.); 2 Group of Genetics, Breeding and Biochemistry of Brassicas, Misión Biológica de Galicia (CSIC) Aptdo. 28, 36080 Pontevedra, Spain; psoengas@mbg.csic.es (P.S.); ecartea@mbg.csic.es (E.C.); pvelasco@mbg.csic.es (P.V.)

**Keywords:** chemoprevention, docetaxel, drug-sensitization, isothiocyanates, prostate cancer, synergism

## Abstract

Glucosinolate-degradation products (GS-degradation products) are believed to be responsible for the anticancer effects of cruciferous vegetables. Furthermore, they could improve the efficacy and reduce side-effects of chemotherapy. The aim of the present study was to determine the cytotoxic effects of GS-degradation products on androgen-insensitive human prostate cancer (AIPC) PC-3 and DU 145 cells and investigate their ability to sensitize such cells to chemotherapeutic drug Docetaxel (DOCE). Cells were cultured under growing concentrations of allyl-isothiocyanate (AITC), sulforaphane (SFN), 4-pentenyl-isothiocyanate (4PI), iberin (IB), indole-3-carbinol (I3C), or phenethyl-isothiocyanate (PEITC) in absence or presence of DOCE. The anti-tumor effects of these compounds were analyzed using the trypan blue exclusion, apoptosis, invasion and RT-qPCR assays and confocal microscopy. We observed that AITC, SFN, IB, and/or PEITC induced a dose- and time-dependent cytotoxic effect on PC-3 and DU 145 cells, which was mediated, at least, by apoptosis and cell cycle arrest. Likewise, we showed that these GS-degradation products sensitized both cell lines to DOCE by synergic mechanisms. Taken together, our results indicate that GS-degradation products can be promising compounds as co-adjuvant therapy in prostate cancer.

## 1. Introduction

Prostate cancer (PC) remains the most commonly diagnosed malignancy for men and the second leading cause of cancer-related deaths for men due to its ability to metastasize [1]. Currently, taxane drugs such as docetaxel (DOCE), are indicated for both the treatment of recurrent hormone-sensitive PC in combination with androgen deprivation therapy (ADT) and treatment of castration-resistant prostate cancer (CRPC) [2]. However, since taxanes have limited effectiveness as well as high toxicity [3,4], there is an urgent need for new treatment strategies in order to try to solve these drawbacks.

Over the past several decades, bioactive constituents of plants such as vegetables of the family Brassicaceae (also called Cruciferae), have gained considerable appreciation [5]. The cruciferous family includes many vegetables that are found in this diet (see Table 1) and whose consumption is beneficial at least by their anticancer effects [6,7]. Recently, it is known that such effects are attributed to glucosinolates (GSs), and more specifically to their degradation products (GS-degradation products): isothiocyanates (ITCs, for example: sulforaphane (SFN), allyl-isothiocyanate (AITC), phenethyl-isothiocyanate (PEITC), iberin (IB), 4-pentenyl-isothiocyanate (4PI)), indoles (for example indole-3-carbinol (I3C)), nitriles, and epithionitriles [7]. Thus ITCs, the most studied GS-degradation products, have been shown to reduce the growth of PCs by regulating target enzymes, controlling apoptosis, inhibiting cell migration and angiogenesis, or blocking the cell cycle [8]. However, the ability of GS-degradation products to reduce dose-limiting toxicity of chemotherapeutic drugs, to increase their efficacy and/or reverse the insensitivity of cancer cells to them has been scarcely studied [9,10].

The aim of this study was to examine in vitro anti-cancer effects of six GS-degradation products (SFN, AITC, PEITC, 4PI, IB, and I3C) in two cell lines of PC, in the absence or presence of the chemotherapeutic drug DOCE, using primary cultures of healthy human prostate epithelial cells (PEC) and the androgen-insensitive human prostate cancer (AIPC) cell lines PC-3 and DU 145. We have observed that ITCs could be effective as co-adjuvant therapy of DOCE, this suggests that cruciferous vegetables could be recommended together with classical chemotherapy agents in patients with CRPC.

## 2. Results

### 2.1. Inhibition of PC Cells Proliferation by GS Degradation-Products and/or DOCE

The doses of GS degradation-products were chosen according to the toxicity levels on PEC cells. Doses up to 20 μM of AITC, 4 μM of PEITC, 500 μM of 4PI, 30 μM of SFN, 30 μM of IB, and 400 μM of I3C μM decreased survival drastically (differences *p* < 0.05).

**Figure 1:**
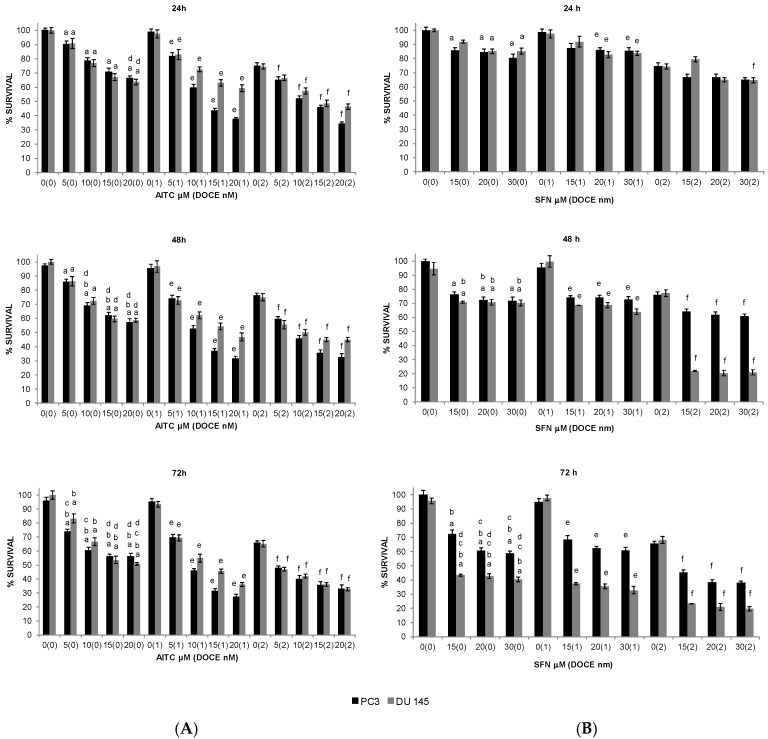
Time-course and dose-response of allyl-isothiocyanate (**A**) and sulforaphane (**B**) treatment effects on proliferation of PC-3 (black pillar) and DU 145 (grey pillar) cells as determined by trypan blue dye exclusion assay. Prostate cancer cells were plated, allowed to attach overnight, and treated with dimethyl sulfoxide (DMSO) (control) or desired concentration of allyl-isothiocyanate (AITC) or sulforaphane (SFN) and/or DOCE (docetaxel) for specified time intervals. Both floating and adherent cells were collected and used for counting of dead and live cells. Data are shown as the mean ± SD of three independent experiments. ^a^, *p* < 0.05, ITC treatment alone significantly different compared with control treatment; ^b^, *p* < 0.05, ITC treatment alone for 48 or 72 h significantly different compared with ITC treatment alone for 24 h; ^c^, *p* < 0.05, ITC treatment alone for 72 h significantly different compared with ITC treatment alone for 48 h; ^d^, *p <* 0.05, ITC treatment alone significantly different compared with 2 nM DOCE alone; ^e^, *p* < 0.05, combination treatment (ITC + DOCE 1 nM) significantly different compared with 1 nM DOCE alone; ^f^, *p* < 0.05, combination treatment (ITC + DOCE 2 nM) significantly different compared with 2 nM DOCE alone (one-way ANOVA).

**Figure 2:**
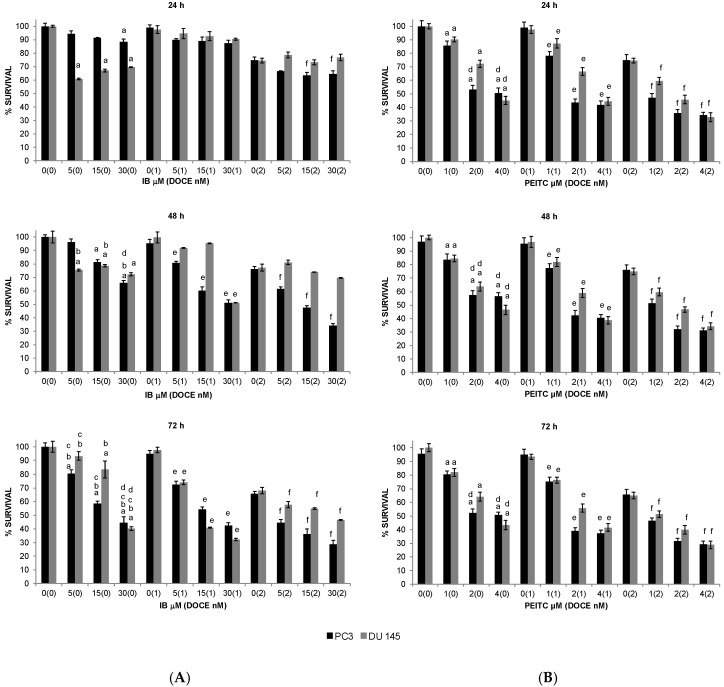
Time-course and dose-response of iberin (**A**) and phenethyl-isothiocyanate (**B**) treatment effects on proliferation of PC-3 (black pillar) and DU 145 (grey pillar) cells as determined by trypan blue dye exclusion assay. Prostate cancer cells were plated, allowed to attach overnight, and treated with DMSO (control) or desired concentration of iberin (IB), phenethyl-isothiocyanate (PEITC) and/or docetaxel (DOCE) for specified time intervals. Both floating and adherent cells were collected and used for counting of dead and live cells. Data are shown as the mean ± SD of three independent experiments. ^a^, *p* < 0.05, ITC treatment alone significantly different compared with control treatment; ^b^, *p* < 0.05, ITC treatment alone for 48 or 72 h significantly different compared with ITC treatment alone for 24 h; ^c^, *p* < 0.05, ITC treatment alone for 72 h significantly different compared with ITC treatment alone for 48 h; ^d^, *p* < 0.05, ITC treatment alone significantly different compared with 2 nM DOCE alone; ^e^, *p* < 0.05, combination treatment (ITC + DOCE 1nM) significantly different compared with 1 nM DOCE alone; ^f^, *p* < 0.05, combination treatment (ITC + DOCE 2nM) significantly different compared with 2 nM DOCE alone (one-way ANOVA).

All the GS degradation-products tested, except I3C and 4PI, reduced the survival of PC-3 and DU 145 cells. AITC, SFN, and IB inhibited the survival of both cell lines in a concentration- and time-dependent manner (Figure 1 and Figure 2A), whereas PEITC inhibited their survival only in a concentration-dependent manner (Figure 2B).

The inhibitory effect induced by AITC was similar for both cell lines (Figure 1A), however the other compounds (Figure 1B and Figure 2) exhibited cell-specific effects or had a higher inhibitory effect on one of the cell lines. For example, the inhibition induced by the SFN treatment was more pronounced on DU 145 cells (57–60%) than on PC-3 cells (28–42%) at 72 h (Figure 1B).

All the GS degradation-products tested (Figure 1 and Figure 2), except SFN on PC-3 cells (Figure 1B), were more effective on PC-3 and DU 145 cells than DOCE. For example, at the maximum dose tested: 20 μM of AITC (Figure 1A) at 24, 48, and 72 h reduced PC-3 and DU 145 viability by ~35%, ~40%, and ~48%; 4 μM of PEITC (Figure 2B) at 24, 48, and 72 h reduced PC-3 and DU 145 cell viability by ~49% and ~55%, ~44% and ~54%, and ~49 and ~57% whereas that 2 nM DOCE alone inhibited the growth of these cells by ~25%, ~24%, and ~34%, respectively.

### 2.2. Sensitization of PC Cells to Growth Suppression by DOCE

The combination therapy with ITCs and DOCE was significantly more efficacious against viability of PC cells compared with ITCs or DOCE treatment alone. This effect was observed for both cell lines tested, so the ITC-mediated sensitization to growth suppression by DOCE was not a cell line-specific response. Analyzing results by the method previously described [11], in general, it was observed that the enhancement of anti-proliferative activity of DOCE mediated by ITC on PC-3 and DU 145 cells can be explained by synergic mechanisms (expected survival rate/observed survival rate >1) (Table A1, Table A2, Table A3, Table A4, Table A5, Table A6, Table A7 and Table A8); only no synergistic effect was observed for SFN on PC-3 cells at 24 h and 48 h and on DU 145 cells at 24 h (Table A3 and Table A4), and for IB on DU 145 cells at 24 and 48 h (Table A6).

### 2.3. Apoptosis

Apoptotic effects were only studied for those compounds which had had some effect on the viability of PC cells, i.e., AITC, SFN, IB, and PEITC.

**Figure 3:**
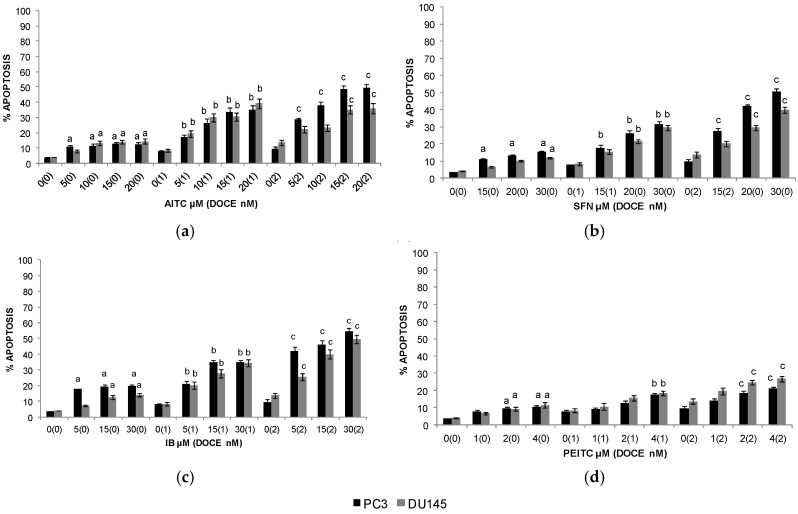
Quantitation of apoptotic PC-3 (black pillar) and DU 145 (grey pillar) cells (DAPI assay) following 72 h treatment with allyl-isothiocyanate (AITC) (**a**), sulforaphane (SFN) (**b**), iberin (IB) (**c**), phenethyl-isothiocyanate (PEITC) (**d**), and/or docetaxel (DOCE). Data are shown as the mean ± SD of three independent experiments. ^a^, *p* < 0.05, ITC (isothiocyanate) treatment alone significantly different compared with control treatment; ^b^, *p* < 0.05, combination treatment (ITC + DOCE 1 nM) significantly different compared with low-dose DOCE alone; ^c^, *p* < 0.05, combination treatment (ITC + DOCE 2 nM) significantly different compared with high-dose DOCE alone (one-way ANOVA).

AITC-, IB-, SFN-, and PEITC-mediated death of PC-3 and DU 145 cells can be explained by apoptotic mechanisms (19.7% and 14.09%, 15.32% and 11.83%, 12.3% and 14.43%, and 10.31% and 11.39% of apoptotic cells with the highest dose of IB, SFN, AITC, and PEITC, for PC-3 and DU 145 cells, respectively). When these cell lines were treated with DOCE, the percentages of apoptotic cells were of up to ~9% and ~14% for PC-3 and DU 145 cells, respectively. Combination therapy with ITC increased the efficacy of chemotherapeutic drug in both cell lines (Figure 3A).

### 2.4. Cytopathic Changes

**Figure 4:**
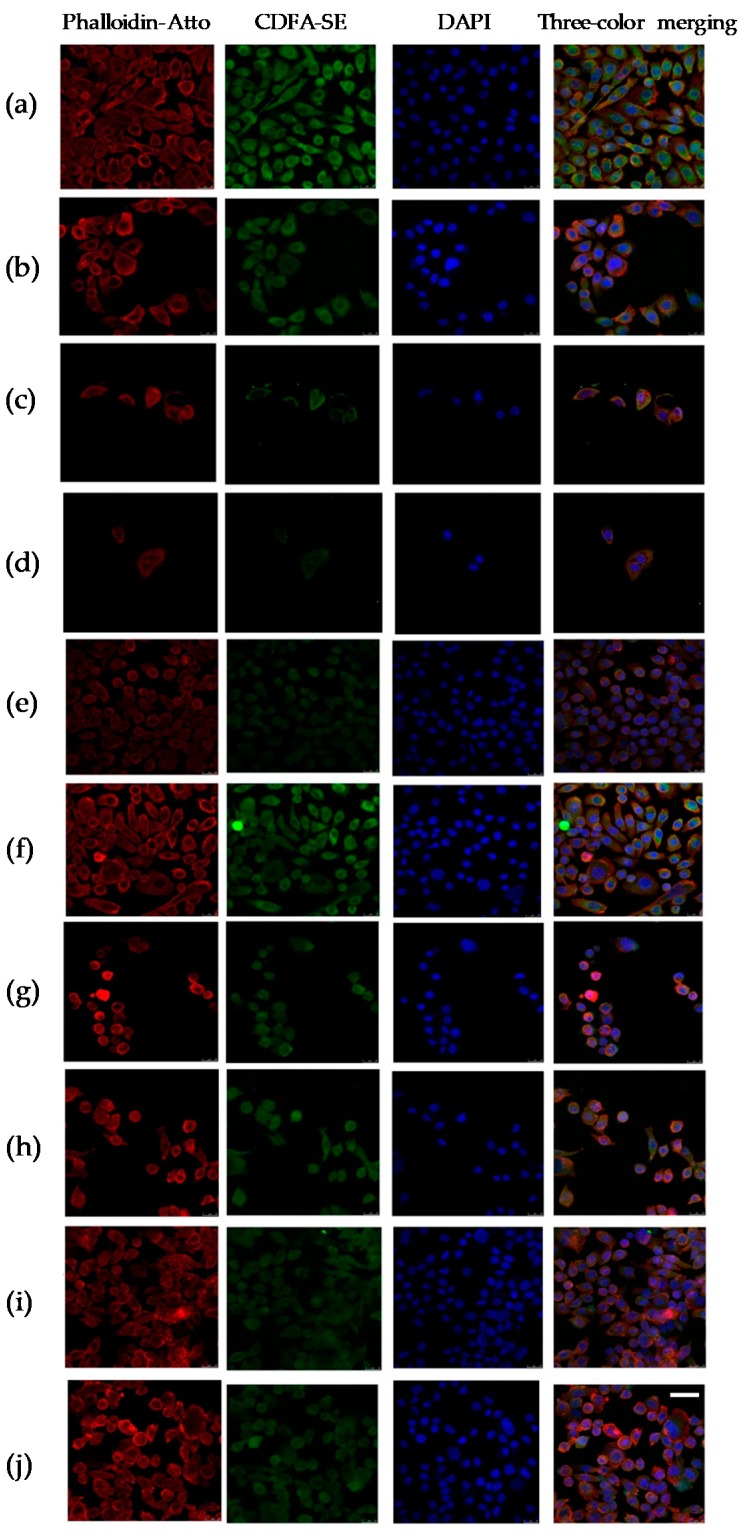
Cytopathic changes in PC-3 cells induced by the control treatment (**a**) or the treatment with DOCE 2 nM (**b**); AITC 20 µM (**c**); AITC 20 µM + DOCE 2 nM (**d**); SFN 30 µM (**e**); SFN 30 µM + DOCE 2 nM (**f**); IB 30 µM (**g**); IB 30 µM + DOCE 2 nM (**h**); PEITC 4 µM (**i**); PEIT and C 4 µM + DOCE 2 nM (**j**). Confocal images show green (carboxyfluorescein diacetate succinimidyl ester, CFDA-SE), red (Phalloidin-Atto 647N), blue (4′, 6-diamidino-2-phenylindole, DAPI) and merge of three channels. The cells treated with AITC (20 µM), SFN (30 µM), IB (30 µM), PEITC (4 µM) and/or DOCE (2 nM) showed a predominantly rounded shape phenotype with DNA condensation. Scale bar = 100 µm.

To explore the role of AITC, IB, SFN, and PEITC in cell damage in PC cells, we examined cytopathic changes in PC-3 cells. In the control group, PC-3 cells showed elongated morphology, ellipsoid nuclei with euchromatin and heterochromatin, actin filaments localized mainly beneath the plasma membrane and cytoplasm stained homogenously with CFDA-SE. The cells treated with AITC 20 µM, IB 30 µM, SFN 30 µM, PEITC 4µM, and/or DOCE 2 nM showed a rounded shape as the predominant phenotype and actin skeleton reorganization (Figure 4).

### 2.5. Migration of PC Cells

**Figure 5:**
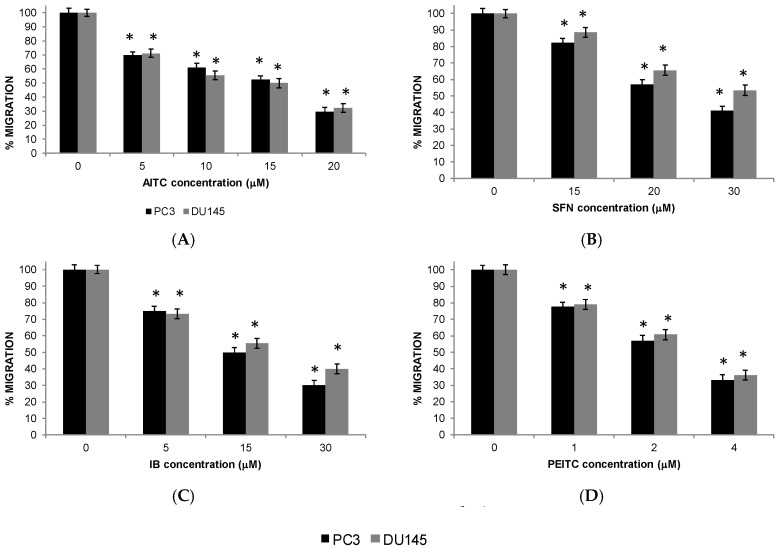
Isothiocyanates inhibit PC-3 (black pillar) and DU 145 (grey pillar) cell migration. Prostate cancer cells were treated with allyl-isothiocyanate (AITC), sulforaphane (SFN), iberin (IB), and phenethyl-isothiocyanate (PEITC). Data are shown as the mean ± SD of three independent experiments. * Isothiocyanate treatment significantly different compared with control treatment (*p* < 0.05).

The migration ability of PC-3 and DU 145 cells was affected by the treatment with ITCs (Figure 5). After the treatment for 72 h the migration ability of PC-3 and DU 145 cells was decreased.

### 2.6. RT-qPCR

**Figure 6:**
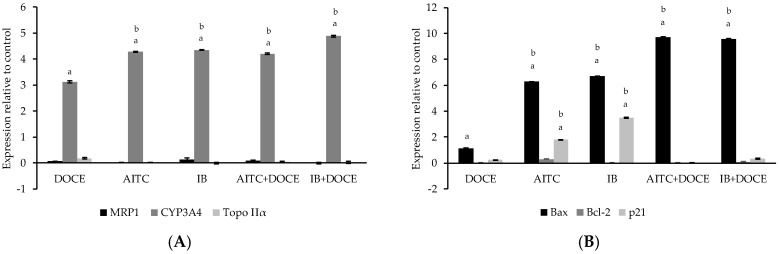
Effect of allyl-isothiocyanate (AITC), iberin (IB) and/or docetaxel (DOCE) on gene expression (2^−∆∆*C*t^) of: (**A**) MRP1, CYP3A4 and Topo IIα; (**B**) Bax, Bcl-2 and p21. Data are shown as the mean ± SD of three independent experiments. Values higher than one were considered positive in comparison to cells treated with control. ^a^ treatment significantly different compared with control (*p* < 0.05). ^b^ treatment significantly different compared with DOCE (*p* < 0.05).

Since AITC and IB were the ITCs that offered the best results as anti-tumor therapy, concretely on PC-3 cells, further studies were focused on this highly metastatic cell line. We verified by RT-q PCR the molecular mechanisms that could be involved in the anti-tumor effects of AITC and IB. The results for the induction drug transporter—(MRP1), drug metabolism—(CYP3A4), target—(DNA topoisomerase II, Topo IIα), migration—(CYP3A4), cell cycle/apoptosis-related (Bax, Bcl2, and p21) genes are shown in Figure 6. Values higher than one were considered positive in comparison to cells treated with control.

The cells treated with AITC, IB, DOCE, DOCE-AITC, and DOCE-IB did not show significant induction of MRP1 or Topo IIα in relation to control or DOCE. On the contrary, all the treatments significantly increased the expression of CYP3A4 as well as the Bax/Bcl-2 expression ratio in PC-3 cells. In relation to the p21, its expression was only significantly modified compared to control cells and cells treated with DOCE by the ITCs, AITC, and IB.

## 3. Discussion

Recently, there is no satisfactory treatment for PC when the cancer cells lose responsiveness to ADT [12]. DOCE is a therapeutic alternative but with important side effects and limited anti-cancer response, among other aspects due to the appearance of resistance [3,4]. These disadvantages could be addressed by combination therapies. In the present study, we observed that ITCs have very promising anti-cancer efficacy on clinically relevant metastatic prostate cells and that they could be used as co-adjuvant therapy in patients with CRPC.

Several ITCs have shown to be potential therapeutic agents for PC since their anti-proliferative [13,14], pro-apoptotic [14,15], and anti-migratory [16,17] effects. Although mechanisms by which they produce these anti-cancer effects are not fully known, their cytotoxic action is usually associated with cell cycle arrest and activation of apoptosis [8,18], as it has been shown in the present study. PC-3 cells treated with AITC and IB increased the expression of p21 and the Bax/Bcl-2 expression ratio, which in turn promotes G1 cell cycle phase arrest [19] and induces the release of cytochrome c causing mitochondrial dysfunction [20]. Collectively, these findings suggest that checkpoints for cell cycle arrest and programmed cell death are regulated by ITCs in PC cells. Therefore, these compounds could play an important role in the management of CRPC. 

**Figure 7:**
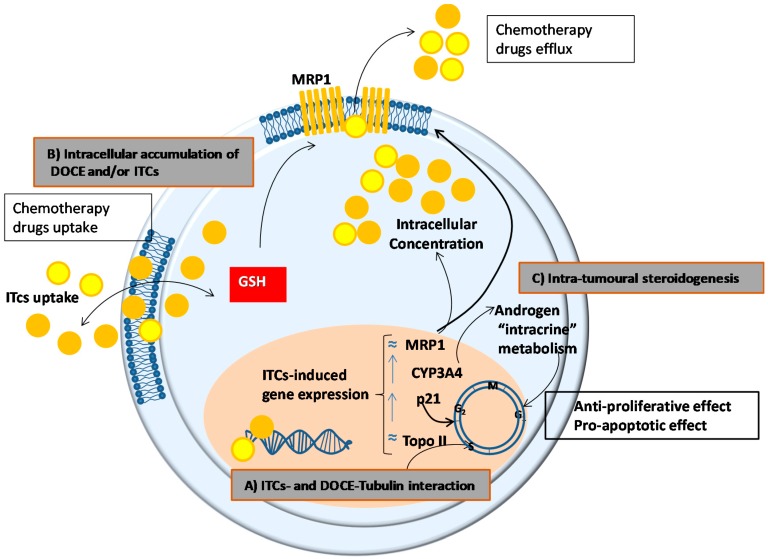
Potential mechanisms to explain the synergistic effect of combined isothiocyanate (ITC, orange circles) and docetaxel (DOCE, yellow circles) in prostate cancer cells. (**A**) Interaction of ITCs and DOCE with the microtubules. (**B**) Uptake, accumulation, and efflux of ITCs and DOCE mediated by the expression of efflux transporters and/or glutathione (GSH) levels/activity. (**C**) Modulation of the intracrine metabolism of androgens. The expression of the genes observed in this study (indicated as ≈ not substantial modification and ↑ increase), produces inhibition of proliferative activity and apoptosis. Abbreviations: MRP1. Multidrug resistance-associated protein 1; p21. Cyclin-dependent kinase inhibitor 1; Topo II. DNA topoisomerase 2-alpha.

More interesting than the anti-cancer effect per se of ITCs was their ability to potentiate the action of DOCE. As it has been previously commented, we found that the combination of DOCE with AITC, PEITC, IB or SFN significantly caused a synergistic sensitization of PC-3 and DU 145 for DOCE-induced apoptosis and DOCE-induced cell growth inhibition. The synergistic effect that we observed could be due to (Figure 7):

(a) A common mechanism of action for ITCs and DOCE at the microtubules. DOCE, a known microtubule-targeting agent, binds to microtubules and causes not only their stabilization but also the inhibition of their depolymerization which exerts anti-proliferative and pro-apoptotic effects, respectively [21]. Likewise, some authors claim that the anti-proliferative and pro-apoptotic effects of ITCs are due to the degradation and polymerization of α and β tubulin [22].
(b) A higher intracellular accumulation of DOCE and/or ITCs, since their efficacy depends on their intracellular accumulation which in turn is determined by the balance between uptake and efflux processes. In this sense, and taking into account that ITCs can inhibit cellular export of anticancer agents [23], we studied the expression of the unidirectional efflux transporter MRP1, which plays a role in the development of drug resistance of various types of cancer [24]. However, the expression of MRP1 was not modified by the treatments tested in the present study.
(c) A modulation of the intracrine metabolism of androgens mediated CYP3A4. CYP3A4 increases the bioavailability of growth-promoting androgens to PC cells [25], so it has special relevance in clinical practice. Low levels of expression of CYP3A4 has been associated with poor prognosis in PC patients such as metastasis, high Gleason score, and reduced survival [26]. This mechanism could not explain the anti-proliferative, pro-apoptotic, and anti-migratory effects of AITC or IB on PC-3 cells shown in this study since we used AIPC cells. However, the intra-tumoral steroidogenesis could affect the surrounding tissue and indirectly influence tumour cell behaviour [27]. Concretely, it has been shown that de novo synthesized steroids by cancer cells play an important role in the establishment of metastasis and the induction of castration resistance in PC cells by affecting androgen receptor positive cells in the tumour microenvironment, this is in stroma fibroblasts, smooth muscle cells, endothelial cells, osteoblasts, and inflammatory cells.
(d) The intracellular level/activity of glutathione (GSH). ITC cell uptake and intracellular accumulation are conditioned by the intracellular levels/activity of GSH, since their uptake occurs via binding with cysteine sulfhydryl groups of GSH [18]. Taking into account that high levels/activity of GSH favour the uptake of ITC [18], the higher cytotoxic effect of SFN on PC-3 cells than on DU 145 cells shown in the present study, could be explained by the higher levels of GSH observed in PC-3 in comparison with DU 145 cells [28].

## 4. Materials and Methods

### 4.1. Cell Culture

Tumorigenic CRPC cell lines PC-3 (CRL-1435) and DU 145 (HTB-81) and healthy PEC (PCAS-440-010) were obtained from ATCC (ATCC; Manassas, VA, USA). PC-3 and DU 145 cell lines are derived from bone metastasis of human prostate adenocarcinoma grade IV and brain metastasis of prostate carcinoma, respectively. Cells were cultured according to ATCC’s instructions with F-12K Medium (cat. no. 30-2004, ATCC) and Prostate Epithelial Cell Growth Kit (cat. no. PCAS-440-040, ATCC). Stock solutions of AITC (cat. no. 36682, Sigma-Aldrich, Madrid, CM, Spain), 4PI (cat. no. I0444, TCI; Paris, France), PEITC (cat. no. 253731, Sigma-Aldrich, Madrid, CM, Spain), SFN (cat. no. S4441, Sigma-Aldrich, Madrid, CM, Spain), IB (cat. no. ab141944, abcam, Cambridge, United Kingdom), I3C (cat. no. I7256, Sigma-Aldrich, Madrid, CM, Spain) and DOCE (cat. no. 01885; Sigma-Aldrich, Madrid, CM, Spain) were prepared in dimethyl sulfoxide (DMSO); cat. no. D2650; Sigma-Aldrich, Madrid, CM, Spain) and diluted with complete medium. An equal volume of DMSO (final concentration < 0.05%) was added to the controls.

### 4.2. Cell Viability Assay

Effect of AITC, 4PI, PEITC, SFN, IB, I3C and/or DOCE treatments on viability of PC-3 and DU 145 cells was determined by trypan blue dye exclusion assay as described previously [29]. Briefly, cells (5 × 10^3^) were seeded in six-well plates, and allowed to attach by overnight incubation. The medium was changed by fresh complete medium containing desired concentrations of AITC (5, 10, 15 and 20 μM), 4PI (10, 25, 50, 100, 500 μM), PEITC (1, 2, 4 μM), SFN (15, 20, 30 μM), IB (5, 15, 30 μM), I3C (100, 160, 285, 400 μM) and/or DOCE (1, 2 nM). Following incubation at 37 °C in a humidified atmosphere of 95% air and 5% CO_2_ for 24, 48 or 72 h, both floating and adherent cells were collected and suspended in 25 mL of PBS (cat. no. D8537; Sigma-Aldrich, Madrid, CM, Spain), mixed with 5 mL of 0.4% trypan blue solution (cat. no. T6146; Sigma-Aldrich, Madrid, CM, Spain) and counted under an inverted microscope (Olympus, Tokyo, Japan).

### 4.3. Determination of Apoptosis

Apoptosis induction in control (DMSO treated) and AITC-, PEITC-, SFN-, IB, and/or DOCE-treated PC cells was assessed by fluorescence after staining with 4′, 6-diamidino-2-phenylindole (DAPI, cat. no. D9542; Sigma-Aldrich, Madrid, MD, Spain) [29]. DAPI is known to form fluorescent complexes with double-stranded DNA. Briefly, 2 × 10^4^ cells were seeded on coverslips and allowed to attach by overnight incubation. Then, cells were treated with DMSO, ITCs (AITC, PEITC, SFN, and IB) and/or DOCE at the same concentrations that were used to evaluate cellular viability. Following incubation at 37 °C for 72 h, the cells were washed with PBS, fixed with 3% paraformaldehyde for 1 h at room temperature, washed again with PBS, and permeabilized with 1% Triton X-100 (cat. no. 142314.1611142314.1611; Panreac, Barcelona, CAT, Spain) for 4 min. Finally and after washing with PBS, the cells were stained with 1 μg/mL DAPI for 5 min and observed under fluorescence microscope at 40× magnification. The apoptotic cells (with condensed and fragmented DNA) were counted. Minimum 300 cells were examined for each treatment, and the percentage of cells with apoptotic signs was calculated.

### 4.4. Cytophatic Changes

PC cells (1 × 10^4^ cells/well) were seeded in growth chambers (cat. no. C6932; Sigma-Aldrich, Madrid, MD, Spain) and incubated for 24 h at 37 °C in a humidified atmosphere of 95% air and 5% CO_2_. Then, the medium was replaced with fresh complete medium containing AITC (20 μM), SFN (30 μM), IB (30 μM), PEITC (4 μM) and/or DOCE (2 nM). After 72 h, PC-3 cells were stained according to previously published procedures [30]. Firstly, the treatments were replaced by PBS solution containing fluorescent dye CFDA-SE (Carboxyfluorescein diacetate succinimidyl ester, cat. no. 1351201EDU; Bio-Rad Laboratories, Madrid, MD, Spain) at 1 mM. After 15 min, the cells were washed with PBS, fixed with 70% ethanol for 5 min, washed again with PBS and stained with Phalloidin-ATTO 1:500 (Phalloidin-ATTO 647N, cat. no. 65906; Sigma-Aldrich, Madrid, MD, Spain). After 1 h, the cells were washed with sodium chloride (NaCl, cat. no. S7653; Sigma-Aldrich Madrid, MD, Spain) at 0.9% and stained with DAPI at 1 μg/mL for 10 min. Finally, slides were embedded in Vecta Shield antifade mounting medium (cat. no. H-1000; Vector Laboratories, Burlingame, CA, USA) and cells were analyzed under a confocal microscope (Leica TCS SP5 X microscope, Leica Microsystems, Wetzlar, Germany).

### 4.5. In Vitro Invasion Assay

The invasion ability of PC-3 and DU 145 cells treated with AITC, PEITC, SFN, or IB, was detected using Millicell Cell Culture Inserts (cat. no. PIEP12R48; Merck Millipore, Madrid, MD, Spain) for 24-well plates [31]. Briefly, cells (1 × 10^5^) were suspended in 100 μL serum-free medium and added into the upper chamber. The lower chamber was filled with 600 μL complete medium containing 10% fetal bovine serum (FBS; Life Technologies, Madrid, MD, Spain). Following incubation at 37 °C in a humidified atmosphere of 95% air and 5% CO_2_ for 72 h, the cells on the upper surface of the membranes were removed by wiping with a cotton swab. Finally, the membranes were fixed with 4% paraformaldehyde for 20 min and stained with crystal violet (cat. no. C6158; Sigma-Aldrich Madrid, MD, Spain) for 15 min. The migrated cells were counted under a microscope (Olympus, Tokyo, Japan) in five randomly selected fields. At least three membranes from three different experiments were analyzed.

### 4.6. Reverse Transcription-quantitative Polymerase Chain Reaction (RT-qPCR)

Drug transporter- (multidrug resistance-associated protein-1, MRP1), drug metabolism- (CYP3A4), target- (DNA topoisomerase II, TopoIIα), migration- (CYP3A4), cell cycle/apoptosis-related (Bax, Bc2, p21) genes were analyzed. PC cells (1 × 10^4^ cells/well) were seeded in growth chambers (cat. no. C6932; Sigma-Aldrich, Madrid, MD, Spain) and incubated for 24 h at 37 °C in a humidified atmosphere of 95% air and 5% CO_2_. Then, the medium was replaced with fresh complete medium containing AITC (20 μM), SFN (30 μM), IB (30 μM), PITC (4 μM) and/or DOCE (2 nM). After 72 h, total RNA was extracted from PC-3 cells™ RNAspin Mini RNA Isolation Kit (Sigma-Aldrich) in accordance with the manufacturer’s protocol. cDNAs were synthesized using a ReadyScript™ cDNA Synthesis Mix (Sigma-Adrich). Real-time PCR reactions were performed using Fast Start SYBR Green Master (BioRad, Hercules, CA, USA). Reaction conditions were as follows: preheated at 95 °C for 3 min, 35 cycles of 94 °C for 30 s, 55–65 °C for 30 s and 72 °C for 40 s followed by 72 °C for 10 min. Quantification was done using the 2^−∆∆*C*t^ method, which calculates the relative changes in gene expression of the target normalized to GAPDH. Primer sequences are listed in Table 2.

### 4.7. Statistical Analysis

The results represent the mean of three independent experiments (mean ± SD). Statistical analysis was conducted using the SPSS software package for Windows, version 21. Analysis between groups was carried out by using one way ANOVA. Difference was considered significant at *p* ≤ 0.05.

## 5. Conclusions

The findings of the present study offer an encouraging perspective for the research of new approaches in the treatment of PC, with the natural compounds as key elements of combination therapies.

## Figures and Tables

**Table 1:** Glucosinolates (GSs) in vegetable and salad crops of Brassicaceae family.

Common Name	Scientific Name	Main GSs	GSs Type
Broccoli	*Brassica oleracea* var. *italica*	Glucoraphanin, Sinigrin, Glucobrassicin	Aliphatic and indolic
Cauliflower	*B. oleracea* var. *botrytis*	Sinigrin, Glucoraphanin, Glucoiberin, Glucobrassicin	Aliphatic and indolic
Brussels sprouts	*B. oleracea* var. *gemmifera*	Sinigrin, Progoitrin, Glucoraphanin, Glucoiberin, Glucobrassicin	Aliphatic and indolic
Cabbage	*B. oleracea* var. *capitata*	Sinigrin, Glucoiberin, Progoitrin, Glucobrassicin	Aliphatic and indolic
Kale	*B. oleracea* var. *acephala*	Sinigrin, Glucoiberin, Glucobrassicin	Aliphatic and indolic
Chinese cabbage	*B. oleracea* var. *pekinensis*	Sinigrin, Progoitrin, Glucobrassicin	Aliphatic and indolic
Kohlrabi	*B. oleracea* var. *gongylodes*	Gluconapin, Glucoerucin, Glucoraphanin, Glucobrassicin	Aliphatic and indolic
Turnip	*Brassica rapa*	Gluconapin, Glucobrassicanapin	Aliphatic
Rutabaga	*Brassica napus* var. *napobrassica*	Sinigrin, Gluconapin, Progoitrin, Glucoerucin, Gluconasturtiin	Aliphatic and aromatic
Nabicol	*B. napus* var. *pabularia*	Glucobrassicanapin, Progoitrin	Aliphatic
Mustard black	*Brassica nigra*	Sinigrin, Gluconapin, Gluconasturtiin	Aliphatic
Mustard brown	*Brassica juncea*	Sinigrin, Progoitrin, Gluconapin, Glucobrassicanapin	Aliphatic
Mustard white	*Sinapis alba*	Glucosinalbin	Aliphatic
Garden cress	*Lepidium sativum*	Glucotropaeolin	Aromatic
Watercress	*Nasturtium officinale*	Glucoiberin, Glucobrassicin, Gluconasturtiin	Aliphatic, indolic and aromatic
Rocket	*Eruca sativa* *Diplotaxis* sp.	Glucoerucin, Glucoraphanin	Aliphatic
Radish	*Raphanus sativa*	Sinigrin, Glucoerucin, Glucotropaeolin, Gluconasturtiin, Glucobrassicin	Aliphatic, indolic and aromatic
Horseradish	*Armoracia lapathifolia*	Sinigrin, Gluconapin, Gluconasturtiin	Aliphatic and aromatic

The GSs consist of a β-thioglucose moiety, a sulfonated oxime moiety, and a variable side chain derived from an amino acid. Based on their amino acid precursors, GSs are classified into three major groups: aliphatic, aromatic, and indolic GSs.

**Table 2:** Sequences of the primers used in reverse transcription-quantitative PCR assays.

Gen/Gene-Related Mechanism		Primes RT-qPCR (5′–3′)
MRP1 Drug-transporter gene		F: TGTGGACGCTCAGAGGTTCA R: CATCGCCATCACAGCATTG
CYP3A4 Drug-metabolism gene Migration gen		F: GGGAAGCAGAGACAGGCAA R: AAGGGGTCTTGTGGATTGTTG
Topo IIα Target genes		F: ATTCAGAGGGGATATGATTCGG R: GGTTAAATACCAAAGGGGCATA
Cell cycle related genes/apoptosis- related genes	Bax	F: AGGATGCGTCCACCAAGAAG R: TGAAGTTGCCGTCAGAAAACA
	Bcl-2	F: ATGTGTGTGGAGAGCGTCAACC R: TGAGCAGAGTCTTCAGAGACAGCC
	p21	F: CCCGTGAGCGATGGAACT R: CGAGGCACAAGGGTACAAGA
GAPDH		F: GAAGACTGTGGATGGCCCCTC R: GTTGAGGGCAATGCCAGCCCC

## Abbreviations

4PI	4-pentenyl-isothiocyanate
ADT	Androgen deprivation therapy
AIPC	Androgen-insensitive human prostate cancer
AITC	Allyl-isothiocyanate
CRPC	Castration-resistant prostate cancer
DOCE	Docetaxel
FBS	Fetal bovine serum
GS	Glucosinolates
GS-degradation products	Glucosinolates-degradation products
GSH	Glutathione
I3C	Indole-3-carbinol
IB	Iberin
ITC	Isothiocyanate
PC	Prostate cancer
PEC	Prostate epithelial cells
PEITC	Phenethyl-isothiocyanate
SFN	Sulforaphane

## Appendix A

**Table A1:** Analysis of synergy between AITC and DOCE calculated by survival rate for PC-3 cells.

	Docetaxel			AITC			Combination Treatment			Index ^5^
	Dose	MSR ^1^	*p*^2,^*	Dose	MSR ^1^	*p*^2,^*	Expected ^3^	Observed ^4^	*p* ^2,♦^	
**24 h**	1	0.99	>0.05	5	0.9	<0.05	0.89	0.82	>0.05	1.09
	1	0.99	>0.05	10	0.78	<0.05	0.77	0.6	<0.05	1.28
	1	0.99	>0.05	15	0.71	<0.05	0.7	0.44	<0.05	1.59
	1	0.99	>0.05	20	0.66	<0.05	0.65	0.37	<0.05	1.76
	2	0.75	<0.05	5	0.9	<0.05	0.68	0.65	>0.05	1.05
	2	0.75	<0.05	10	0.78	<0.05	0.59	0.52	>0.05	1.13
	2	0.75	<0.05	15	0.71	<0.05	0.53	0.46	<0.05	1.15
	2	0.75	<0.05	20	0.66	<0.05	0.5	0.34	<0.05	1.47
**48 h**	1	0.95	>0.05	5	0.86	<0.05	0.82	0.74	>0.05	1.11
	1	0.95	>0.05	10	0.69	<0.05	0.66	0.53	<0.05	1.25
	1	0.95	>0.05	15	0.62	<0.05	0.59	0.37	<0.05	1.59
	1	0.95	>0.05	20	0.57	<0.05	0.54	0.31	<0.05	1.74
	2	0.76	<0.05	5	0.86	<0.05	0.65	0.59	>0.05	1.1
	2	0.76	<0.05	10	0.69	<0.05	0.52	0.46	>0.05	1.13
	2	0.76	<0.05	15	0.62	<0.05	0.47	0.35	<0.05	1.34
	2	0.76	<0.05	20	0.57	<0.05	0.43	0.32	<0.05	1.34
**72 h**	1	0.95	>0.05	5	0.74	<0.05	0.7	0.69	>0.05	1.01
	1	0.95	>0.05	10	0.6	<0.05	0.57	0.46	<0.05	1.24
	1	0.95	>0.05	15	0.56	<0.05	0.53	0.31	<0.05	1.71
	1	0.95	>0.05	20	0.56	<0.05	0.53	0.27	<0.05	1.96
	2	0.66	<0.05	5	0.74	<0.05	0.49	0.48	>0.05	1.02
	2	0.66	<0.05	10	0.6	<0.05	0.4	0.4	>0.05	1
	2	0.66	<0.05	15	0.56	<0.05	0.37	0.35	>0.05	1.06
	2	0.66	<0.05	20	0.56	<0.05	0.37	0.33	>0.05	1.12

Abbreviations: AITC, allyl isothiocyanate; DMSO, dimethyl sulfoxide; DOCE, docetaxel; MSR, Mean survival rate. ^1^ MSR of treated group/DMSO-treated control group. ^2^ *p* value was determined by one-way ANOVA followed by Tukey’s test. ^3^ Survival rate of DOCE group multiplied by survival rate of the AITC group. ^4^ Survival rate of combination treatment group (DOCE + AITC). ^5^ Index was calculated by dividing the expected survival rate by the observed survival rate. An index of >1 indicates synergistic effect and an index of <1 indicates less than additive effect. * *p* < 0.05, significantly different compared with control treatment. ^♦^ *p* < 0.05, comparison between expected and observed survival rate.

**Table A2:** Analysis of synergy between AITC and DOCE calculated by survival rate for DU 145 cells.

	Docetaxel			AITC			Combination Treatment			Index ^5^
	Dose	MSR ^1^	*p*^2,^*	Dose	MSR ^1^	*p*^2,^*	Expected ^3^	Observed ^4^	*p* ^2,♦^	
**24 h**	1	0.98	>0.05	5	0.91	<0.05	0.89	0.83	>0.05	1.07
	1	0.98	>0.05	10	0.77	<0.05	0.75	0.73	>0.05	1.03
	1	0.98	>0.05	15	0.67	<0.05	0.66	0.63	>0.05	1.05
	1	0.98	>0.05	20	0.64	<0.05	0.63	0.59	>0.05	1.07
	2	0.75	<0.05	5	0.91	<0.05	0.68	0.67	>0.05	1.01
	2	0.75	<0.05	10	0.77	<0.05	0.58	0.57	>0.05	1.02
	2	0.75	<0.05	15	0.67	<0.05	0.5	0.49	>0.05	1.02
	2	0.75	<0.05	20	0.64	<0.05	0.48	0.46	>0.05	1.04
**48 h**	1	0.97	>0.05	5	0.86	<0.05	0.83	0.73	<0.05	1.14
	1	0.97	>0.05	10	0.72	<0.05	0.7	0.62	<0.05	1.13
	1	0.97	>0.05	15	0.6	<0.05	0.58	0.54	>0.05	1.07
	1	0.97	>0.05	20	0.59	<0.05	0.57	0.47	<0.05	1.21
	2	0.75	<0.05	5	0.86	<0.05	0.65	0.56	<0.05	1.16
	2	0.75	<0.05	10	0.72	<0.05	0.54	0.5	>0.05	1.08
	2	0.75	<0.05	15	0.6	<0.05	0.45	0.45	>0.05	1
	2	0.75	<0.05	20	0.59	<0.05	0.44	0.45	>0.05	0.98
**72 h**	1	0.93	>0.05	5	0.83	<0.05	0.77	0.69	<0.05	1.12
	1	0.93	>0.05	10	0.67	<0.05	0.62	0.55	<0.05	1.13
	1	0.93	>0.05	15	0.54	<0.05	0.5	0.46	>0.05	1.09
	1	0.93	>0.05	20	0.51	<0.05	0.47	0.36	<0.05	1.31
	2	0.65	<0.05	5	0.83	<0.05	0.54	0.47	<0.05	1.15
	2	0.65	<0.05	10	0.67	<0.05	0.44	0.42	>0.05	1.05
	2	0.65	<0.05	15	0.54	<0.05	0.35	0.36	>0.05	0.97
	2	0.65	<0.05	20	0.51	<0.05	0.33	0.33	>0.05	1

Abbreviations: AITC, allyl isothiocyanate; DMSO, dimethyl sulfoxide; DOCE, docetaxel; MSR, Mean survival rate. ^1^ MSR of treated group/DMSO-treated control group. ^2^ *p* value was determined by one-way ANOVA followed by Tukey’s test. ^3^ Survival rate of DOCE group multiplied by survival rate of the AITC group. ^4^ Survival rate of combination treatment group (DOCE + AITC). ^5^ Index was calculated by dividing the expected survival rate by the observed survival rate. An index of >1 indicates synergistic effect and an index of <1 indicates less than additive effect. * *p* < 0.05, significantly different compared with control treatment. ^♦^ *p* < 0.05, comparison between expected and observed survival rate.

**Table A3:** Analysis of synergy between SFN and DOCE calculated by survival rate for PC-3 cells.

	Docetaxel			SFN			Combination Treatment			Index ^5^
	Dose	MSR ^1^	*p*^2,^*	Dose	MSR ^1^	*p*^2,^*	Expected ^3^	Observed ^4^	*p* ^2,♦^	
**24 h**	1	0.99	>0.05	15	0.86	<0.05	0.85	0.87	>0.05	0.98
	1	0.99	>0.05	20	0.85	<0.05	0.84	0.86	>0.05	0.98
	1	0.99	>0.05	30	0.81	<0.05	0.8	0.86	>0.05	0.93
	2	0.75	<0.05	15	0.86	<0.05	0.65	0.67	>0.05	0.97
	2	0.75	<0.05	20	0.85	<0.05	0.64	0.67	>0.05	0.96
	2	0.75	<0.05	30	0.81	<0.05	0.61	0.65	>0.05	0.94
**48 h**	1	0.95	>0.05	15	0.76	<0.05	0.72	0.74	>0.05	0.97
	1	0.95	>0.05	20	0.72	<0.05	0.68	0.74	>0.05	0.92
	1	0.95	>0.05	30	0.72	<0.05	0.68	0.73	>0.05	0.93
	2	0.76	<0.05	15	0.76	<0.05	0.58	0.64	<0.05	0.91
	2	0.76	<0.05	20	0.72	<0.05	0.55	0.62	<0.05	0.89
**72 h**	1	0.95	>0.05	15	0.72	<0.05	0.68	0.68	>0.05	1
	1	0.95	>0.05	20	0.61	<0.05	0.58	0.62	>0.05	0.94
	1	0.95	>0.05	30	0.59	<0.05	0.56	0.61	>0.05	0.92
	2	0.66	<0.05	15	0.72	<0.05	0.48	0.45	>0.05	1.07
	2	0.66	<0.05	20	0.61	<0.05	0.4	0.39	>0.05	1.03
	2	0.66	<0.05	30	0.59	<0.05	0.39	0.38	>0.05	1.03

Abbreviations: DMSO, dimethyl sulfoxide; DOCE, docetaxel; MSR, mean survival rate; SFN, sulforaphane. ^1^ MSR of treated group/DMSO-treated control group. ^2^ *p* value was determined by one-way ANOVA followed by Tukey’s test. ^3^ Survival rate of DOCE group multiplied by survival rate of the SFN group. ^4^ Survival rate of combination treatment group (DOCE + SFN). ^5^ Index was calculated by dividing the expected survival rate by the observed survival rate. An index of >1 indicates synergistic effect and an index of <1 indicates less than additive effect. * *p* < 0.05, significantly different compared with control treatment. ^♦^ *p* < 0.05, comparison between expected and observed survival rate.

**Table A4:** Analysis of synergy between SFN and DOCE calculated by survival rate for DU 145 cells.

	Docetaxel			SFN			Combination Treatment			Index ^5^
	Dose	MSR ^1^	*p*^2,^*	Dose	MSR ^1^	*p*^2,^*	Observed ^4^	MSR ^1^	*p* ^2,♦^	
**24 h**	1	0.98	>0.05	15	0.92	>0.05	0.9	0.92	>0.05	0.98
	1	0.98	>0.05	20	0.85	<0.05	0.83	0.83	>0.05	1
	1	0.98	>0.05	30	0.85	<0.05	0.83	0.84	>0.05	0.99
	2	0.75	<0.05	15	0.92	>0.05	0.69	0.79	<0.05	0.87
	2	0.75	<0.05	20	0.85	<0.05	0.64	0.65	>0.05	0.98
	2	0.75	<0.05	30	0.85	<0.05	0.64	0.65	>0.05	0.98
**48 h**	1	1	>0.05	15	0.71	<0.05	0.71	0.69	>0.05	1.03
	1	1	>0.05	20	0.71	<0.05	0.71	0.69	>0.05	1.03
	1	1	>0.05	30	0.7	<0.05	0.7	0.64	>0.05	1.09
	2	0.77	<0.05	15	0.71	<0.05	0.55	0.22	<0.05	2.5
	2	0.77	<0.05	20	0.71	<0.05	0.55	0.2	<0.05	2.75
**72 h**	1	0.98	>0.05	15	0.43	<0.05	0.42	0.37	>0.05	1.14
	1	0.98	>0.05	20	0.43	<0.05	0.42	0.36	>0.05	1.17
	1	0.98	>0.05	30	0.4	<0.05	0.39	0.33	>0.05	1.18
	2	0.68	<0.05	15	0.43	<0.05	0.29	0.23	>0.05	1.26
	2	0.68	<0.05	20	0.43	<0.05	0.29	0.21	>0.05	1.38
	2	0.68	<0.05	30	0.4	<0.05	0.27	0.2	>0.05	1.35

Abbreviations: DMSO, dimethyl sulfoxide; DOCE, docetaxel; MSR, mean survival rate; SFN, sulforaphane. ^1^ MSR of treated group/DMSO-treated control group. ^2^ *p* value was determined by one-way ANOVA followed by Tukey’s test. ^3^ Survival rate of DOCE group multiplied by survival rate of the SFN group. ^4^ Survival rate of combination treatment group (DOCE + SFN). ^5^ Index was calculated by dividing the expected survival rate by the observed survival rate. An index of >1 indicates synergistic effect and an index of <1 indicates less than additive effect. * *p* < 0.05, significantly different compared with control treatment. ^♦^ *p* < 0.05, comparison between expected and observed survival rate.

**Table A5:** Analysis of synergy between IB and DOCE calculated by survival rate for PC-3 cells.

	Docetaxel			IB			Combination Treatment			Index ^5^
	Dose	MSR ^1^	*p*^2,^*	Dose	MSR ^1^	*p*^2,^*	Expected ^3^	Observed ^4^	*p* ^2,♦^	
**24 h**	1	0.99	>0.05	5	0.94	>0.05	0.93	0.9	>0.05	1.03
	1	0.99	>0.05	15	0.91	>0.05	0.9	0.89	>0.05	1.01
	1	0.99	>0.05	30	0.89	<0.05	0.88	0.88	>0.05	1
	2	0.75	<0.05	5	0.94	>0.05	0.71	0.66	>0.05	1.08
	2	0.75	<0.05	15	0.91	>0.05	0.68	0.64	>0.05	1.06
	2	0.75	<0.05	30	0.89	<0.05	0.67	0.65	>0.05	1.03
**48 h**	1	0.95	>0.05	5	0.96	>0.05	0.91	0.81	<0.05	1.12
	1	0.95	>0.05	15	0.81	<0.05	0.77	0.6	<0.05	1.28
	1	0.95	>0.05	30	0.66	<0.05	0.63	0.51	<0.05	1.24
	2	0.76	<0.05	5	0.96	>0.05	0.73	0.62	<0.05	1.18
	2	0.76	<0.05	15	0.81	<0.05	0.62	0.48	<0.05	1.29
	2	0.76	<0.05	30	0.66	<0.05	0.5	0.34	<0.05	1.47
**72 h**	1	0.95	>0.05	5	0.81	<0.05	0.77	0.73	>0.05	1.05
	1	0.95	>0.05	15	0.58	<0.05	0.55	0.54	>0.05	1.02
	1	0.95	>0.05	30	0.45	<0.05	0.43	0.42	>0.05	1.02
	2	0.66	<0.05	5	0.81	<0.05	0.53	0.45	>0.05	1.18
	2	0.66	<0.05	15	0.58	<0.05	0.38	0.36	>0.05	1.06
	2	0.66	<0.05	30	0.45	<0.05	0.3	0.29	>0.05	1.03

Abbreviations: DMSO, dimethyl sulfoxide; DOCE, docetaxel; IB, iberin; MSR, mean survival rate. ^1^ MSR of treated group/DMSO-treated control group. ^2^ *p* value was determined by one-way ANOVA followed by Tukey’s test. ^3^ Survival rate of DOCE group multiplied by survival rate of the IB group. ^4^ Survival rate of combination treatment group (DOCE + IB). ^5^ Index was calculated by dividing the expected survival rate by the observed survival rate. An index of >1 indicates synergistic effect and an index of <1 indicates less than additive effect. * *p* < 0.05, significantly different compared with control treatment. ^♦^ *p* < 0.05, comparison between expected and observed survival rate.

**Table A6:** Analysis of synergy between IB and DOCE calculated by survival rate for DU 145 cells.

	Docetaxel			IB			Combination Treatment			Index ^5^
	Dose	MSR ^1^	*p*^2,^*	Dose	MSR ^1^	*p*^2,^*	Expected ^3^	Observed ^4^	*p* ^2,♦^	
**24 h**	1	0.98	>0.05	5	0.61	<0.05	0.6	0.95	<0.05	0.63
	1	0.98	>0.05	15	0.67	<0.05	0.66	0.93	<0.05	0.71
	1	0.98	>0.05	30	0.7	<0.05	0.69	0.9	<0.05	0.77
	2	0.75	<0.05	5	0.61	<0.05	0.46	0.79	<0.05	0.58
	2	0.75	<0.05	15	0.67	<0.05	0.5	0.73	<0.05	0.68
	2	0.75	<0.05	30	0.7	<0.05	0.53	0.77	<0.05	0.69
**48 h**	1	1	>0.05	5	0.75	<0.05	0.75	0.92	<0.05	0.82
	1	1	>0.05	15	0.79	<0.05	0.79	0.95	<0.05	0.83
	1	1	>0.05	30	0.72	<0.05	0.72	0.51	<0.05	1.41
	2	0.77	<0.05	5	0.75	<0.05	0.58	0.81	<0.05	0.72
	2	0.77	<0.05	15	0.79	<0.05	0.61	0.74	<0.05	0.82
	2	0.77	<0.05	30	0.72	<0.05	0.55	0.7	<0.05	0.79
**72 h**	1	0.98	>0.05	5	0.93	>0.05	0.91	0.74	<0.05	1.23
	1	0.98	>0.05	15	0.84	<0.05	0.82	0.41	<0.05	2
	1	0.98	>0.05	30	0.4	<0.05	0.39	0.32	>0.05	1.22
	2	0.68	<0.05	5	0.93	>0.05	0.63	0.58	>0.05	1.09
	2	0.68	<0.05	15	0.84	<0.05	0.57	0.55	>0.05	1.04
	2	0.68	<0.05	30	0.4	<0.05	0.27	0.47	<0.05	0.57

Abbreviations: DMSO, dimethyl sulfoxide; DOCE, docetaxel; IB, iberin; MSR, mean survival rate. ^1^ MSR, of treated group/DMSO-treated control group. ^2^ *p* value was determined by one-way ANOVA followed by Tukey’s test. ^3^ Survival rate of DOCE group multiplied by survival rate of the IB group. ^4^ Survival rate of combination treatment group (DOCE + IB). ^5^ Index was calculated by dividing the expected survival rate by the observed survival rate. An index of >1 indicates synergistic effect and an index of <1 indicates less than additive effect. * *p* < 0.05, significantly different compared with control treatment. ^♦^ *p* < 0.05, comparison between expected and observed survival rate.

**Table A7:** Analysis of synergy between PEITC and DOCE calculated by survival rate for PC-3 cells.

	Docetaxel			PEITC			Combination Treatment			Index ^5^
	Dose	MSR ^1^	*p*^2,^*	Dose	MSR ^1^	*p*^2,^*	Expected ^3^	Observed ^4^	*p* ^2,♦^	
**24 h**	1	0.99	>0.05	1	0.86	<0.05	0.85	0.78	>0.05	1.09
	1	0.99	>0.05	2	0.53	<0.05	0.52	0.44	<0.05	1.18
	1	0.99	>0.05	4	0.51	<0.05	0.5	0.42	<0.05	1.19
	2	0.75	<0.05	1	0.86	<0.05	0.65	0.47	<0.05	1.38
	2	0.75	<0.05	2	0.53	<0.05	0.4	0.36	>0.05	1.11
	2	0.75	<0.05	4	0.51	<0.05	0.38	0.34	>0.05	1.12
**48 h**	1	0.95	>0.05	1	0.84	<0.05	0.8	0.77	>0.05	1.04
	1	0.95	>0.05	2	0.58	<0.05	0.55	0.42	<0.05	1.31
	1	0.95	>0.05	4	0.56	<0.05	0.53	0.41	<0.05	1.29
	2	0.76	<0.05	1	0.84	<0.05	0.64	0.51	<0.05	1.25
	2	0.76	<0.05	2	0.58	<0.05	0.44	0.32	<0.05	1.38
	2	0.76	<0.05	4	0.56	<0.05	0.43	0.31	<0.05	1.39
**72 h**	1	0.95	>0.05	1	0.81	<0.05	0.77	0.75	>0.05	1.03
	1	0.95	>0.05	2	0.52	<0.05	0.49	0.39	<0.05	1.26
	1	0.95	>0.05	4	0.51	<0.05	0.48	0.37	<0.05	1.3
	2	0.66	<0.05	1	0.81	<0.05	0.53	0.47	<0.05	1.13
	2	0.66	<0.05	2	0.52	<0.05	0.34	0.32	>0.05	1.06
	2	0.66	<0.05	4	0.51	<0.05	0.34	0.29	>0.05	1.17

Abbreviations: DMSO, dimethyl sulfoxide; DOCE, docetaxel; MSR, mean survival rate; PEITC, phenethyl-ITC. ^1^ MSR, of treated group/DMSO-treated control group. ^2^ *p* value was determined by one-way ANOVA followed by Tukey’s test. ^3^ Survival rate of DOCE group multiplied by survival rate of the PEITC group. ^4^ Survival rate of combination treatment group (DOCE + PEITC). ^5^ Index was calculated by dividing the expected survival rate by the observed survival rate. An index of >1 indicates synergistic effect and an index of <1 indicates less than additive effect. * *p* < 0.05, significantly different compared with control treatment. ^♦^ *p* < 0.05, comparison between expected and observed survival rate.

**Table A8:** Analysis of synergy between PEITC and DOCE calculated by survival rate for DU 145 cells.

	Docetaxel			PEITC			Combination Treatment			Index ^5^
	Dose	MSR ^1^	*p*^2,^*	Dose	MSR ^1^	*p*^2,^*	Expected ^3^	Observed ^4^	*p* ^2,♦^	
**24 h**	1	0.98	>0.05	1	0.9	<0.05	0.88	0.87	>0.05	1.01
	1	0.98	>0.05	2	0.72	<0.05	0.71	0.67	>0.05	1.06
	1	0.98	>0.05	4	0.45	<0.05	0.44	0.44	>0.05	1
	2	0.75	<0.05	1	0.9	<0.05	0.68	0.6	>0.05	1.13
	2	0.75	<0.05	2	0.72	<0.05	0.54	0.46	<0.05	1.17
	2	0.75	<0.05	4	0.45	<0.05	0.34	0.33	>0.05	1.03
**48 h**	1	1	>0.05	1	0.85	<0.05	0.85	0.84	>0.05	1.01
	1	1	>0.05	2	0.66	<0.05	0.66	0.61	>0.05	1.08
	1	1	>0.05	4	0.48	<0.05	0.48	0.4	>0.05	1.2
	2	0.77	<0.05	1	0.85	<0.05	0.65	0.61	>0.05	1.07
	2	0.77	<0.05	2	0.66	<0.05	0.51	0.48	>0.05	1.06
	2	0.77	<0.05	4	0.48	<0.05	0.37	0.35	>0.05	1.06
**72 h**	1	0.98	>0.05	1	0.82	<0.05	0.8	0.8	>0.05	1
	1	0.98	>0.05	2	0.67	<0.05	0.66	0.58	<0.05	1.14
	1	0.98	>0.05	4	0.45	<0.05	0.44	0.44	>0.05	1
	2	0.68	<0.05	1	0.82	<0.05	0.56	0.54	>0.05	1.04
	2	0.68	<0.05	2	0.67	<0.05	0.46	0.42	>0.05	1.1
	2	0.68	<0.05	4	0.45	<0.05	0.31	0.3	>0.05	1.03

Abbreviations: DMSO, dimethyl sulfoxide; DOCE, docetaxel; MSR, mean survival rate; PEITC, phenethyl-ITC. ^1^ MSR, of treated group/DMSO-treated control group. ^2^ *p* value was determined by one-way ANOVA followed by Tukey’s test. ^3^ Survival rate of DOCE group multiplied by survival rate of the PEITC group. ^4^ Survival rate of combination treatment group (DOCE + PEITC). ^5^ Index was calculated by dividing the expected survival rate by the observed survival rate. An index of >1 indicates synergistic effect and an index of <1 indicates less than additive effect. * *p* < 0.05, significantly different compared with control treatment. ^♦^ *p* < 0.05, comparison between expected and observed survival rate.
